# Prediction of depression risk in chronic kidney disease using PSO-optimized random forest: a web-based application based on CHARLS data

**DOI:** 10.3389/fpsyg.2026.1787463

**Published:** 2026-08-11

**Authors:** Siyu Li, Xianhua Wang, Jiangli Hu

**Affiliations:** Sichuan Provincial Center for Mental Health, Sichuan Provincial People's Hospital, School of Medicine, University of Electronic Science and Technology of China, Chengdu, China

**Keywords:** CHARLS, chronic kidney disease, depression, intelligent optimization, prediction model

## Abstract

**Background:**

Depression is a prevalent psychological issue among chronic kidney disease (CKD) patients. Such symptoms can greatly affect the physical and mental health and life expectancy of middle-aged and older persons with CKD.

**Objective:**

The aim of this study is to develop a depression risk prediction model for CKD patients, laying the scientific groundwork for early intervention.

**Methods:**

This research utilized data from the 2015 China Health and Retirement Longitudinal Study (CHARLS), a dataset representative of the Chinese population. The study examined 52 indicators, encompassing socio-demographic variables, behavioral factors, health status, and psychological health parameters. Statistical analysis was performed using SPSS 26.0 and Python. LASSO regression was employed to identify independent predictors of depression risk in patients with CKD. Subsequently, a random forest model was selected to construct the depression risk prediction model based on these predictors, followed by model validation. The model was assessed using accuracy, recall, specificity, F1 score, Area Under Curve (AUC) value, and clinical decision curves. A particle swarm optimization (PSO) algorithm from intelligent optimization algorithms was applied to fine-tune the model's hyperparameters, which were then compared against the unoptimized random forest algorithm.

**Results:**

The final analysis included 734 CKD patients from the CHARLS database. A total of 326(44.4%) CKD patients exhibited depressive symptoms. The findings revealed that gender, household registration, place of residence, self-rated health, arthritis, stomach disease, height, life satisfaction, glycated hemoglobin, educational level, instrumental activities of daily living (IADL), and pain are predictive factors for depression in CKD patients. These factors were used to construct the PSO-random forest model, which demonstrated good consistency and accuracy. The predictive model achieved an AUC value of 0.820.

**Conclusion:**

This study presents a reliable PSO-random forest model for predicting depression risk in CKD patients, leveraging CHARLS data and interpretable features. This tool presents a valuable means of early screening and delivering personalized care, with the potential to significantly improve mental health outcomes within this population.

## 1 Introduction

Chronic kidney disease (CKD) refers to kidney structure or function abnormalities lasting for 3 months or more, accompanied by or not accompanied by a decline in glomerular filtration rate (GFR), abnormal kidney pathology, or evidence of kidney damage (abnormalities in blood, urine components, or imaging studies) [Kidney Disease: Improving Global Outcomes (KDIGO) Hepatitis C Work Group, 2018]. An eGFR of less than 60 mL/min per 1.73 m^2^ for 3 months or more, with or without evidence of kidney damage, is indicative of CKD. CKD has become a global public health issue and is one of the leading causes of death from non-communicable diseases worldwide. In China, the prevalence of CKD in adults is 38%. CKD severely affects people's physical health and mental health (Hill et al., 2016), leading to increased hospitalization rates, and a significant decrease in quality of life and life expectancy. The World Health Organization (World Health Organization, 2023) considers depression to be a common mental disorder characterized by sadness, loss of interest or pleasure, feelings of guilt or low self-worth, sleep or appetite disturbances, feelings of fatigue, and difficulty concentrating. It is estimated that 20–25% of the adult population with kidney disease suffer from depression (Oyekçin et al., 2012). CKD patients experience a high burden of somatic symptoms, impaired quality of life, and role impairment that may predispose them to increased risks of depression (Kommer et al., 2025). CKD patients not only endure a significant burden of somatic symptoms but also commonly experience diminished quality of life and impaired social functioning (Casaux-Huertas et al., 2025). These factors collectively constitute critical predisposing elements for increased depression risk. This highlights the existence of unique risk factors within this population that render them more vulnerable to depression. Depression can severely impact both physical and mental health, potentially leading to various adverse outcomes such as falls, frailty, and dementia. Consequently, addressing mental health issues in this demographic requires targeted attention (Liu et al., 2025).

A risk prediction model is a tool that can be used to assess the risk of depression in CKD patients (Wu et al., 2020; Nooripour et al., 2021). Most previous studies (Jang, 2025; Jafari et al., 2025) have focused on investigating the prevalence and influencing factors of depression, with few attempts to develop risk prediction models (Huang et al., 2025). Given the prevalence of depressive symptoms in CKD patients and their complex relationship with kidney function, establishing a predictive model for depression in patients with CKD has significant clinical importance. However, current models are based on the general population, and there have been no reports of predictive models for depression in CKD patients. Therefore, this study aims to construct a predictive model for depression in CKD patients using a random forest model, combined with PSO algorithm optimization of model hyperparameters to improve the model's predictive accuracy. The Shaply Additive Explanations (SHAP) (Kruk, 2010) method is used to identify key influencing factors of depression in CKD patients. Additionally, this study will develop a web-based application platform to translate research findings into practical applications. This will not only help doctors to identify tendencies toward depression in CKD patients early on and take timely intervention measures but also provide patients with personalized health management advice, enhancing their quality of life.

## 2 Methods

### 2.1 Data sources

The data for this study was sourced from the 2015 wave of the CHARLS, a nationwide survey led by the National School of Development at Peking University. Since the project's inception in 2011, four follow-up surveys have been conducted in the years 2013, 2015, 2018, and 2020 (Zhao et al., 2014). This study employed a multi-stage stratified probability proportional to size sampling method, investigating social and medical data of middle-aged and elderly residents aged 45 and above from 28 provinces, autonomous regions, and municipalities across China, making the data representative of the entire country. The follow-ups combined household and health examination surveys to provide foundational data needed for research on China's aging population, aiding in policy formulation and enhancing the quality of life for older people. The study was approved by the Peking University Biomedical Ethics Committee, and all participants provided written informed consent before participating in the study. The ethical application for the collection of human subject data in CHARLS has been approved by the Peking University Biomedical Ethics Committee (IRB0001052-11,015). All research methods were conducted in accordance with the Helsinki Declaration. The inclusion criteria for this study are as follows: (1) participants with a clinical diagnosis of CKD. The diagnosis of CKD was confirmed based on self-reported medically diagnosed kidney disease in the CHARLS questionnaire. (2) patients aged 45 and above. The exclusion criteria are as follows: (1) patients lacking key variables for assessment. In this study, samples with missing key variables were excluded-specifically, data with missing values in the outcome variable (CESD-10) or CKD diagnosis status were removed.

### 2.2 Variable selection

#### 2.2.1 Outcome variable

Depression was selected as the outcome variable in this study. In CHARLS, the Center for Epidemiologic Studies Depression Scale-10 (CESD-10) was used to assess depression among respondents. The scale consists of 10 items rated using a Likert 4-point scale. Items 5 (“I felt hopeful about the future”) and 8 (“I felt happy”) are positive items, while the rest are negative. Negative items are scored as “rarely or none of the time (less than 1 day) = 0,” “some or a little of the time (1–2 days) = 1,” “occasionally or a moderate amount of the time (3–4 days) = 2,” and “most or all of the time (5–7 days) = 3,” while positive items are reverse-scored. The scores of each item are summed to obtain a total score ranging from 0 to 30. A total score of 10 or above is defined as the presence of depressive symptoms, while a total score below 10 is defined as the absence of depressive symptoms. Higher scores indicate more severe depressive symptoms. The CESD-10 scale demonstrates good sensitivity and specificity, with a Cronbach's alpha coefficient ranging from 0.73 to 0.82 and a test-retest reliability coefficient of 0.71 (Andresen et al., 1994).

#### 2.2.2 Demographic variables

Demographic variables include age, gender, marital status, domicile, habiation, education level, retirement status, number of household members, number of surviving children, and insurance status. Marital status is divided into married and unmarried (including divorced). Domicile and habiation are both categorized into non-rural and rural areas. Education level is classified into four categories: illiterate, primary school and below, middle school, and above middle school.

#### 2.2.3 Behavioral factors

Behavioral factors include smoking history, alcohol consumption history, social activities, access to tap water, and sleep duration. Social activities are treated as a count variable, representing the cumulative number of social activities participated in by respondents. Sleep duration is based on the question, “On average, how many hours of actual sleep did you get each night in the past month?”.

#### 2.2.4 Health factors

Based on previous research and clinical expertise, the study selected 13 chronic diseases, including hypertension, dyslipidemia, diabetes, cancer, chronic lung disease, liver disease, heart disease, stroke, stomach disease, mental illness, memory-related disease, and asthma, disability, vision status, hearing status, dental status, cognitive function, activities of daily living (ADL), IADL, pain, history of falls within a year and hospitalization in the past year. The presence of the 13 chronic diseases was determined by the question, “Has a doctor ever told you that you have any of the following chronic conditions?” Disability was assessed based on the question, “Do you have any of the following disability problems?” Any “yes” response to any of the five disability categories was defined as disability. Cognitive function in this study is divided into mental status and episodic memory ability, with a maximum score of 21, indicating stronger cognitive function with higher scores. ADL and IADL in the CHARLS questionnaire consist of 11 items: dressing, bathing, eating, transferring (getting in or out of bed or chairs), toileting, continence, doing housework, cooking, shopping, managing money, and taking medications. Each item has four options: “no difficulty,” “some difficulty but can still do,” “need help,” and “cannot do.” The first two options were coded as 1, and the last two options were coded as 0, with the sums calculated separately. Finally, pain was assessed using the question, “Which parts of your body have you experienced pain in?”.

#### 2.2.5 Physiological and laboratory indicators

Physiological and laboratory indicators include height, weight, waist circumference, serum urea nitrogen, blood glucose, creatinine, total cholesterol, triglycerides, high-density lipoprotein cholesterol (HDL-C), low-density lipoprotein Cholesterol high-density lipoprotein (LDL-C), C-reactive protein (CRP), glycated hemoglobin (HbA1c), and Cystatin C. These indicators were obtained from the physical examination and blood test questionnaires in CHARLS.

#### 2.2.6 Psychological factors

Life satisfaction and self-rated health were selected as psychological factors for inclusion in the study. Both life satisfaction and self-rated health were categorized into five levels for analysis.

### 2.3 Data analysis and model optimization

Data analysis was conducted using SPSS 26.0. Initially, descriptive statistical analysis was performed on continuous variables using the mean and standard deviation, while frequency analysis was conducted on categorical variables using percentages. For count variables, the median and interquartile range were employed for description. Subsequently, comparisons within and between groups were conducted using chi-square tests, independent sample *t*-tests, and non-parametric tests, respectively, with statistical significance set at *P* < 0.001. To identify the most relevant variables for the model, LASSO regression was utilized to screen for independent predictors of depression risk in CKD. Finally, based on the selected independent predictors, a random forest model was chosen to construct the depression risk prediction model, which was then validated. The model was evaluated using accuracy, recall, specificity, F1 score, AUC value, and clinical decision curves.

Intelligent optimization algorithms are a class of optimization methods based on intelligent behaviors in nature, mimicking the behaviors of organisms or populations to solve complex optimization problems. By simulating natural adaptive processes such as evolution, learning, competition, and collaboration, they gradually approximate the optimal solution. After initial model training, we will apply the PSO algorithm from intelligent optimization algorithms to optimize the hyperparameters of the model. The optimized random forest algorithm will then be compared with its pre-optimization version.

### 2.4 Model interpretation

SHAP is an interpretability tool for machine learning based on Shaply values from game theory, designed to quantify the contribution of each feature to the model's prediction outcomes. By calculating the marginal contribution of each feature in different input combinations, SHAP values quantify the importance of each feature to the model's predictions. This is particularly useful in high-dimensional datasets or black-box models. In this study, by plotting SHAP dependence plots, SHAP summary plots, and SHAP waterfall plots for the random forest, we can effectively understand the working mechanism and decision-making process of the model.

### 2.5 Application development

**Figure 1:**
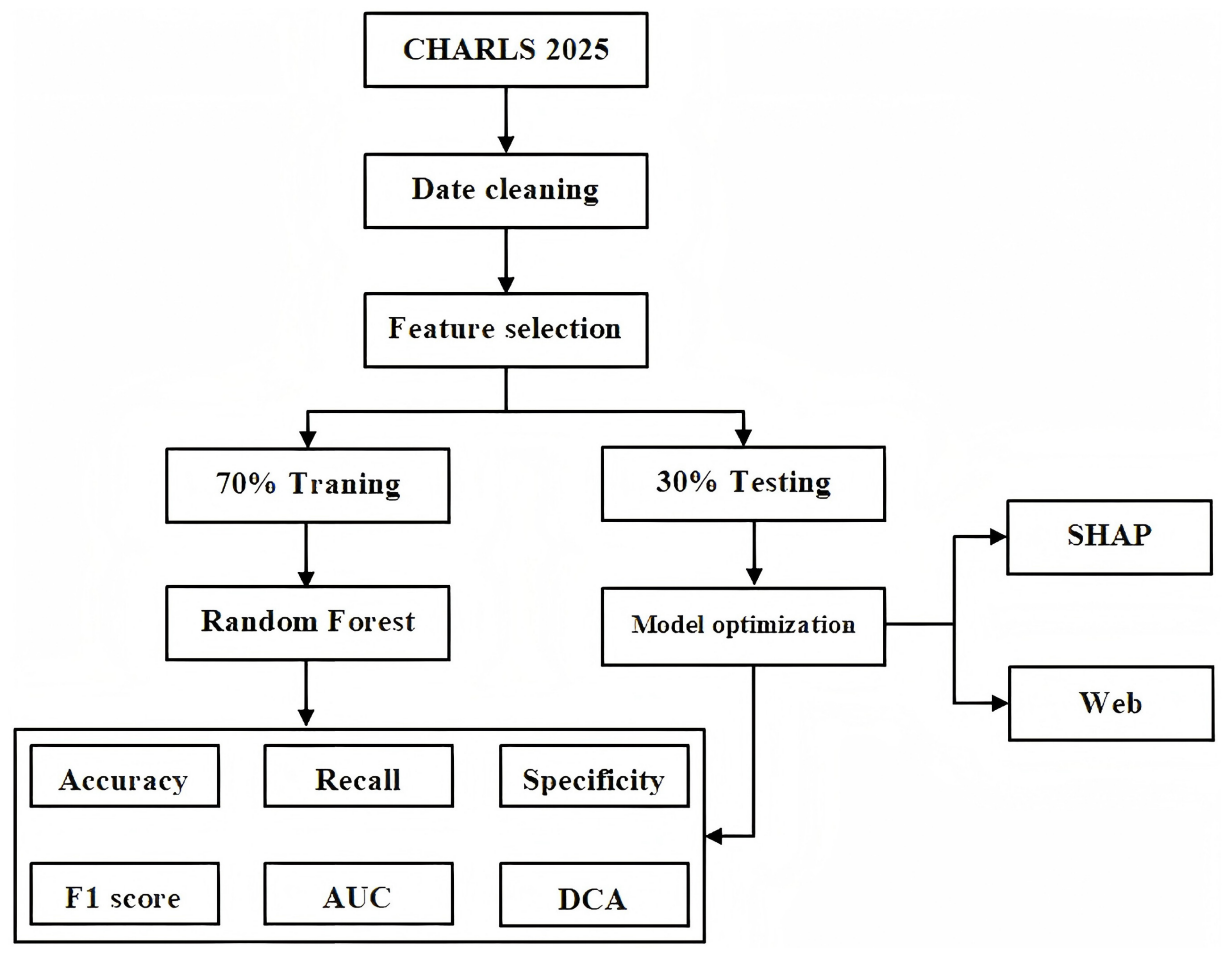
Flowchart of the study.

To better apply the model in clinical practice, we will use Python's Streamlit library to deploy and build a web application for the model. By presenting the model in the form of a web application, we aim to reduce the user threshold, enabling clinicians and patients to quickly obtain individual depression risk predictions and improving the efficiency of clinical work. Additionally, by uploading the web application to the cloud, remote access and sharing become convenient, allowing people to promptly obtain depression assessments. Finally, as new data is input, we will continuously adjust and optimize the web application to enhance the accuracy and practicality of the model. A detailed flowchart of this study is shown in Figure 1.

## 3 Results

### 3.1 Participant characteristics

Initially, the 2015 wave of CHARLS included a total of 20957 respondents. The original 2015 CHARLS dataset included a total of 19,816 middle-aged and elderly participants aged 45 and above. After applying our inclusion and exclusion criteria, we sequentially excluded participants without a diagnosis of CKD, those missing depression assessment data, and those with incomplete key variables. Ultimately, 734 participants were included in the final analysis,among which 326 patients had depressive symptoms, accounting for 44.4%. The demographic and clinical characteristics of the participants are presented in Table 1. Specifically, there were 407 male participants, accounting for 55.4%, and 327 female participants, accounting for 44.6%.

**Table 1:** Participant characteristics of the study population.

Variable	Non-depression	Depression	*P*
Gender			< 0.001
Female	151 (46.2)	176 (53.8)	
Male	257 (63.1)	150 (36.9)	
Education level			< 0.001
Illiterate	127 (43.8)	163 (56.2)	
Primary school	110 (58.2)	79 (41.8)	
Secondary school	101 (62.0)	62 (38.0)	
College or above	70 (76.1)	22 (23.9)	
Pain	0 [0,0]	4 [0,9]	< 0.001
ADL	0 [0,0]	0 [0,1]	< 0.001
IADL	0 [0,0]	0 [0,2]	< 0.001
Social activities	1 [0,2]	1 [0,1]	< 0.001
Number of surviving children	2 [2,3]	2 [2,3]	0.204
Number of household members	3 [2,4]	3 [2,3]	0.388
Cognition function	13 [10.5,14.5]	11.5 [8.5,13.5]	< 0.001
Insurance status			0.538
No	27 (60)	18 (40)	
Yes	381 (55.3)	308 (44.7)	
Dental status			0.700
No	352 (55.9)	278 (44.1)	
Yes	56 (53.8)	48 (46.2)	
Hearing status			< 0.001
Poor or deaf	54 (41.9)	75 (58.1)	
Ordinary	228 (53.8)	196 (46.2)	
Good	73 (70.9)	30 (29.1)	
Very good	44 (66.7)	22 (33.3)	
Excellent	9 (75.0)	3 (25.0)	
Vision status			< 0.001
Poor or blind	76 (42.7)	102 (57.3)	
General	219 (56.2)	171 (43.8)	
Good	64 (65.3)	34 (34.7)	
Very good	43 (72.9)	16 (27.1)	
Excellent	6 (66.7)	3 (33.3)	
History of falls within a year			< 0.001
No	332 (60.1)	220 (39.9)	
Yes	76 (41.8)	106 (58.2)	
Access to tap water			0.035
No	105 (49.5)	107 (50.5)	
Yes	303 (58.0)	219 (42.0)	
Disability			< 0.001
No	263 (62.5)	158 (37.5)	
Yes	145 (46.3)	168 (53.7)	
Life Satisfaction			< 0.001
Not satisfied at all	1 (4.8)	20 (95.2)	
Not quite satisfied	10 (16.7)	50 (83.3)	
Relatively satisfied	216 (53.3)	189 (46.7)	
Very satisfied	152 (72.7)	57 (27.3)	
Extremely satisfied	29 (74.4)	10 (25.6)	
Retirement status			< 0.001
No	316 (52.6)	285 (47.4)	
Yes	92 (69.2)	41 (30.8)	
History of hospitalization within a year			0.001
No	329 (59.0)	229 (41.0)	
Yes	79 (44.9)	97 (55.1)	
Smoking history			0.017
No	179 (51.0)	172 (49.0)	
Yes	229 (59.8)	154 (40.2)	
Drinking history			< 0.001
No	173 (48.6)	183 (51.4)	
Yes	235 (62.2)	143 (37.8)	
Self-rated health			< 0.001
Poor	13 (18.3)	58 (81.7)	
Average	72 (35.5)	131 (64.5)	
Good	248 (66.8)	123 (33.2)	
Very good	45 (81.8)	10 (18.2)	
Excellent	30 (88.2)	4 (11.8)	
Domicile			< 0.001
No	117 (67.2)	57 (32.8)	
Yes	291 (52.0)	269 (48.0)	
Habitation			0.004
No	171 (62.4)	103 (37.6)	
Yes	237 (51.5)	223 (48.5)	
Marital status			0.009
No	36 (42.4)	49 (57.6)	
Yes	372 (57.3)	277 (42.7)	
Hypertension			0.001
No	257 (60.8)	166 (39.2)	
Yes	151 (48.6)	160 (51.4)	
Diabetes			0.027
No	359 (57.3)	268 (42.7)	
Yes	49 (45.8)	58 (54.2)	
Cancer			0.783
No	394 (55.5)	316 (44.5)	
Yes	14 (58.3)	10 (41.7)	
Lung disease			0.021
No	316 (58.1)	228 (41.9)	
Yes	92 (48.4)	98 (51.6)	
Heart attack			< 0.001
No	297 (60.1)	197 (39.9)	
Yes	111 (46.3)	129 (53.8)	
Stroke			0.032
No	386 (56.7)	295 (43.3)	
Yes	22 (41.5)	31 (58.5)	
Mental illness			0.028
No	403 (56.2)	314 (43.8)	
Yes	5 (29.4)	12 (70.6)	
Arthritis			< 0.001
No	187 (69.0)	84 (31.0)	
Yes	221 (47.7)	242 (52.3)	
Dyslipidemia			0.066
No	303 (57.7)	222 (42.3)	
Yes	105 (50.2)	104 (49.8)	
Liver disease			0.018
No	352 (57.5)	260 (45.5)	
Yes	56 (45.9)	66 (54.1)	
Gastrosis			< 0.001
No	246 (64.1)	138 (35.9)	
Yes	162 (46.3)	188 (53.7)	
Asthma			0.301
No	370 (56.2)	288 (43.8)	
Yes	38 (50.0)	38 (50.0)	
Memory-related disease			0.010
No	400 (56.5)	308 (43.5)	
Yes	8 (30.8)	18 (69.2)	
Height	1.597 ± 0.980	1.571 ± 0.990	< 0.001
Weight	61.622 ± 11.910	59.128 ± 12.103	0.005
Waist circumference	86.135 ± 15.126	84.238 ± 13.514	0.077
Sleep	6.298 ± 1.765	5.660 ± 2.133	< 0.001
Blood urea nitrogen	16.053 ± 6.140	16.254 ± 6.201	0.662
Blood glucose	101.117 ± 27.894	102.721 ± 41.004	0.530
Creatinine	0.918 ± 0.630	0.894 ± 0.724	0.738
Total Cholesterol	183.245 ± 33.975	182.668 ± 37.714	0.828
Triglycerides	136.149 ± 79.508	140.931 ± 87.544	0.439
HDL-C	51.451 ± 11.987	51.285 ± 11.280	0.848
LDL-C	102.854 ± 27.380	101.152 ± 28.276	0.410
CRP	3.014 ± 6.667	3.086 ± 7.795	0.893
HbA1c	5.946 ± 0.996	6.015 ± 1.166	0.384
Cystatin C	0.916 ± 0.401	0.911 ± 0.405	0.879

ADL, Activities of Daily Living; IADL, Instrumental Activities of Daily Living; Sleep, nighttime sleep duration; HDL-C, High-Density Lipoprotein Cholesterol; LDL-C, Low-Density Lipoprotein Cholesterol; CRP, C-Reactive Protein; HbA1c, Glycated Hemoglobin.

### 3.2 Feature selection

**Figure 2:**
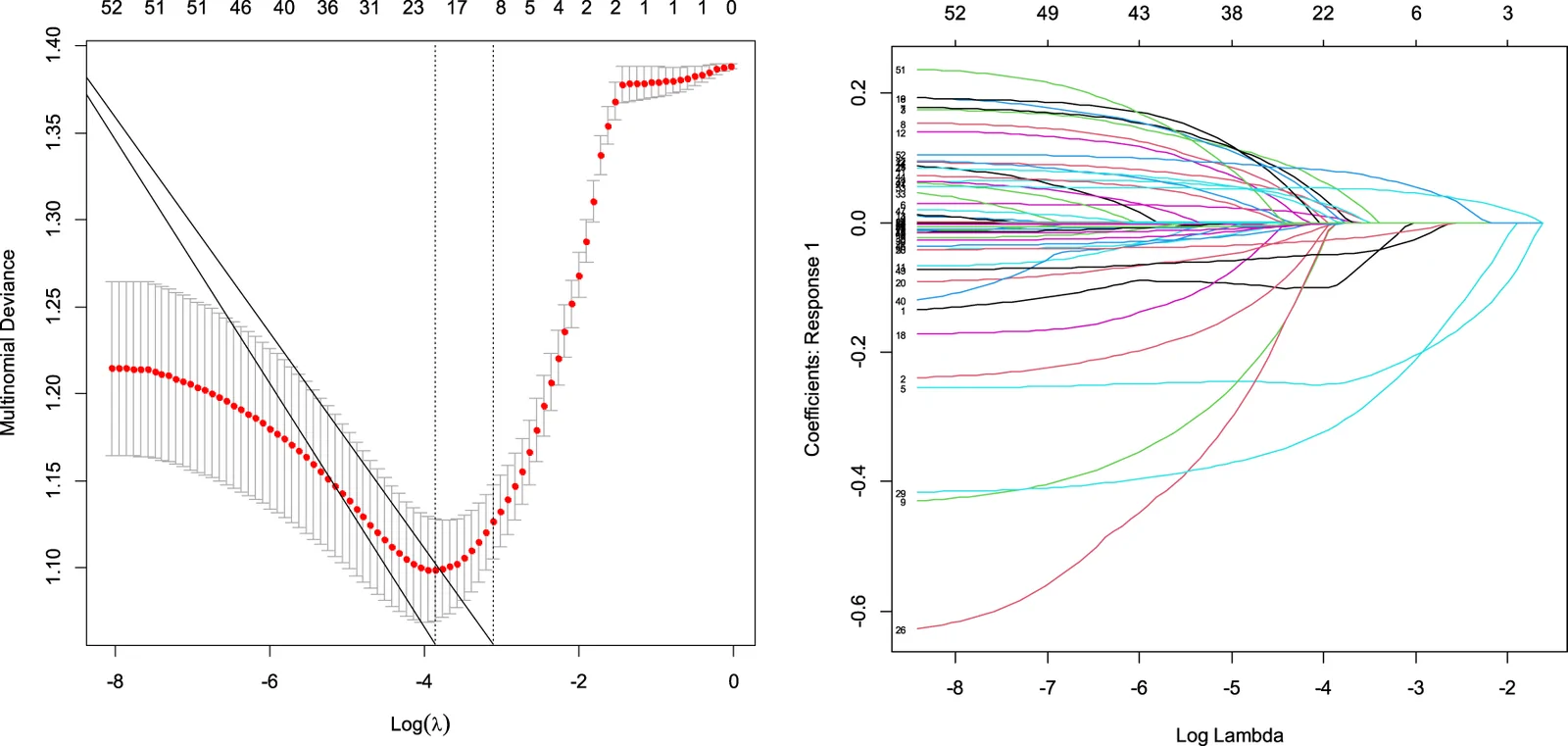
Variable selection diagram for LASSO regression of the depression risk prediction model for CKD patients.

Although random forest possesses built-in capability for assessing feature importance, it may still be affected by redundant features in high-dimensional data. To select variables most closely related to the model. In this study, LASSO was employed for feature selection and linear regularization, while random forest was used to capture non-linear relationships and interactions. This approach aims to reduce feature dimensionality, improve model training efficiency, and provide a clearer set of variables for subsequent SHAP interpretation. This method has been demonstrated in previous medical prediction model studies to enhance model robustness and clinical interpretability (Lin et al., 2022). We did not normalize the data because the study chose the Random Forest algorithm for model training, which is a decision tree-based algorithm insensitive to the scale of the data. By setting depression as the dependent variable and potential influencing factors of depression in patients with chronic kidney disease as independent variables, we conducted model selection using a gradual compression method. By introducing L1 regularization, we could effectively shrink the coefficients of the independent variables. We used 10-fold cross-validation to determine the regularization parameter λ and selected the λ value that minimized the model error as the final optimal parameter. Finally, the included influencing factors were gender, domicile, habitation, self-rated health, arthritis, gastric disease, height, life satisfaction, HbA1c, education level, IADL, and pain. As shown in Figure 2.

### 3.3 Model evaluation and optimization

**Figure 3:**
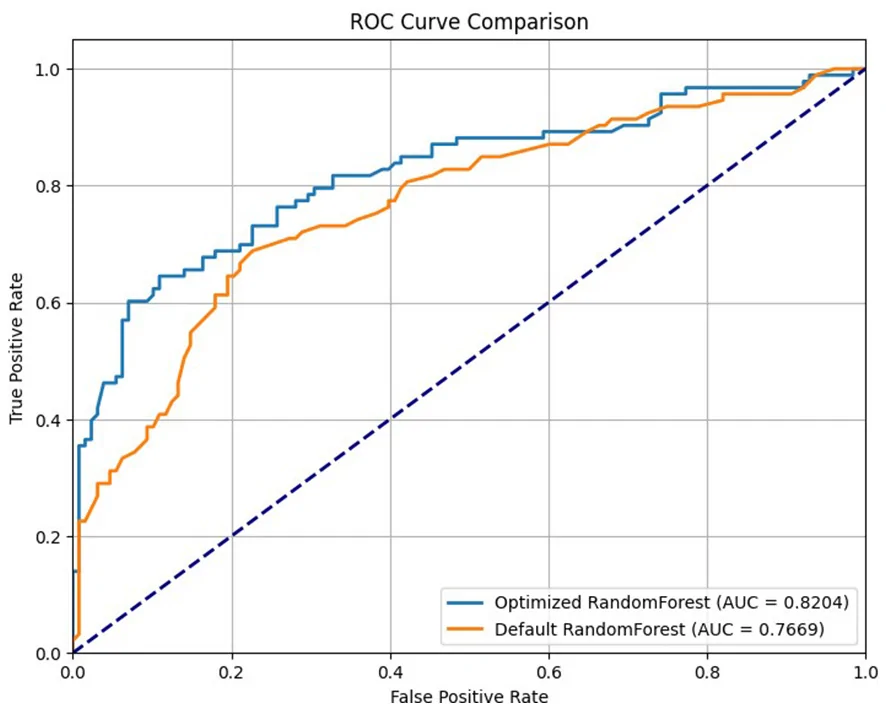
ROC curve of the depression risk prediction model for CKD patients.

**Figure 4:**
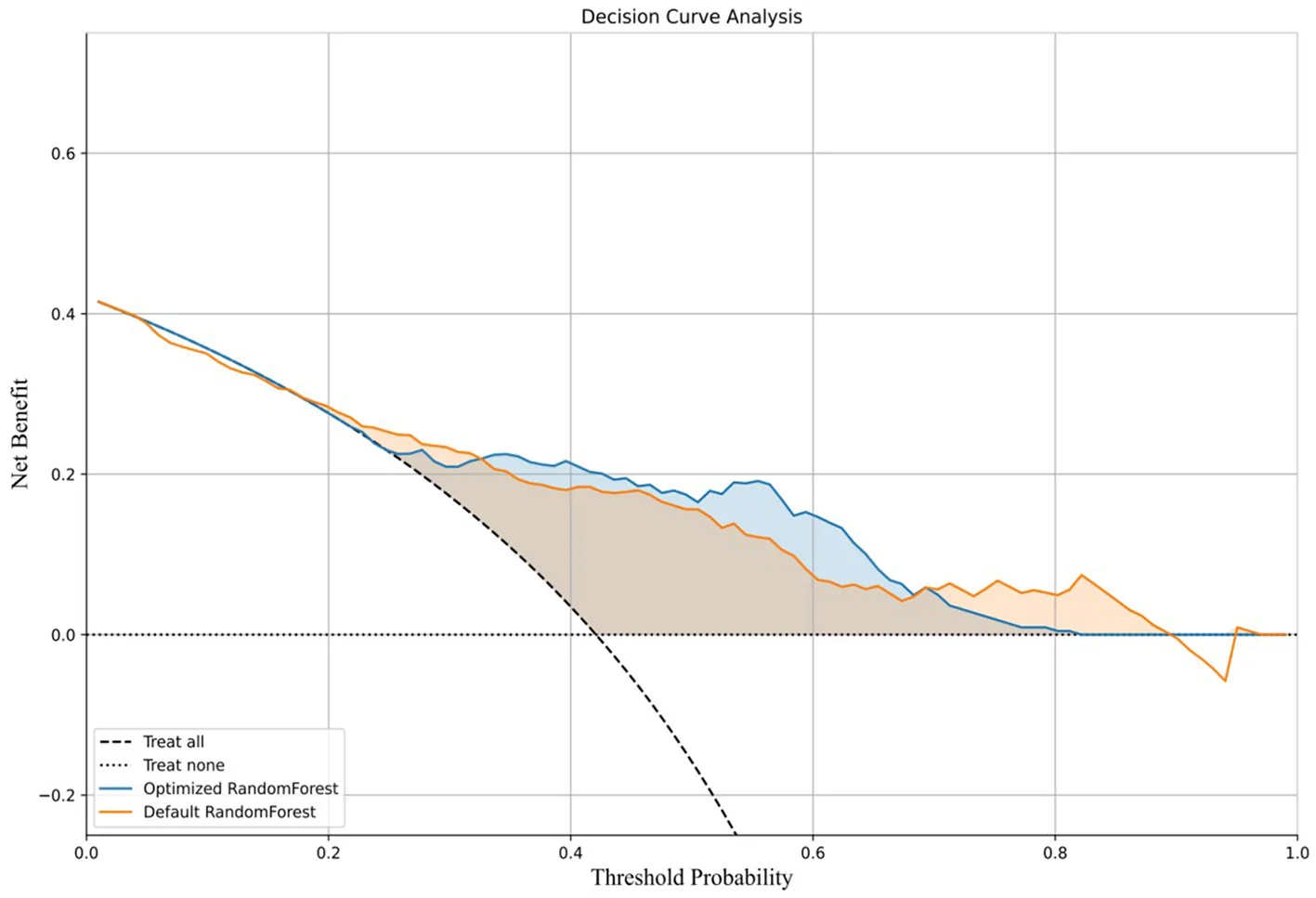
Clinical decision curve of the depression risk prediction model for CKD patients.

After training the model with the relevant variables selected through LASSO regression, the dataset was divided into a training set and a test set in a 70% to 30% ratio. The detailed performance metrics of the model are shown in Table 2. To further enhance the 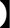 model's performance after its initial construction, we chose the PSO algorithm to optimize the model's hyperparameters. The PSO algorithm was configured as follows: a swarm size of 50, 100 iterations, an inertia weight of 0.8, with the optimization objectives being to maximize the F1 score and AUC. The optimized hyperparameters of the random forest were ultimately determined through a grid search combined with PSO,with detailed indicators presented in Table 2 and Figure 3. It can be observed that the optimized model significantly improved in accuracy and AUC, while also providing higher net benefits across a broader range of thresholds. This indicates that the optimized model has greater practical clinical application value, reducing unnecessary interventions and enhancing prediction accuracy. For a visual representation, see Figure 4.

**Table 2:** Model performance of a depression risk prediction model for CKD patients.

Model	Accuracy	Recall	Specificity	F1 Score	AUC
Random Forest	0.7376	0.6452	0.8047	0.6742	0.7669
PSO-random Forest	0.7511	0.6989	0.7891	0.7027	0.8204

### 3.4 Model interpretation

**Figure 5:**
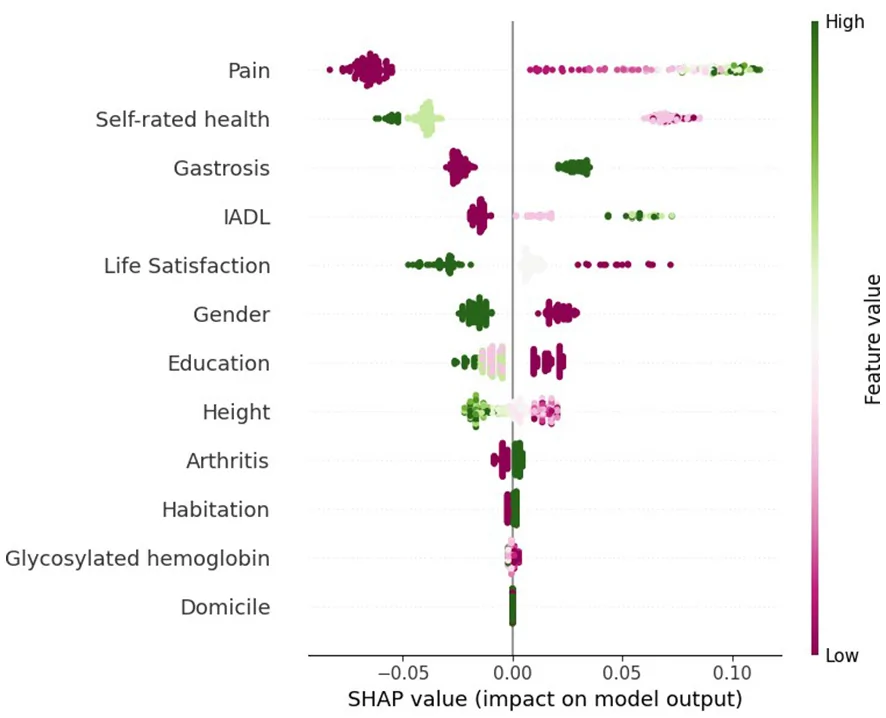
SHAP summary plot of the depression risk prediction model for CKD patients.

**Figure 6:**
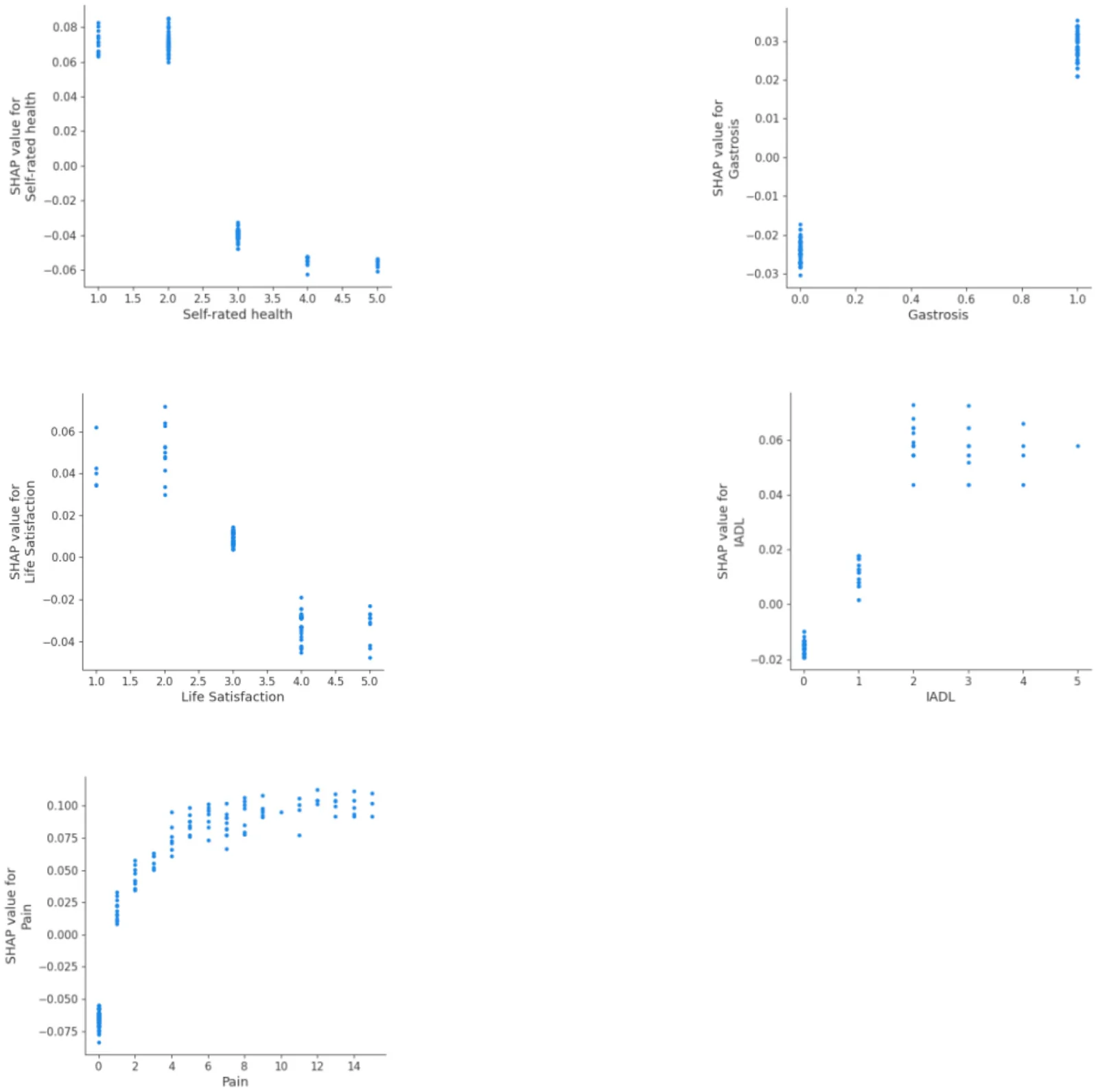
SHAP dependence plot of the depression risk prediction model for CKD patients.

**Figure 7:**
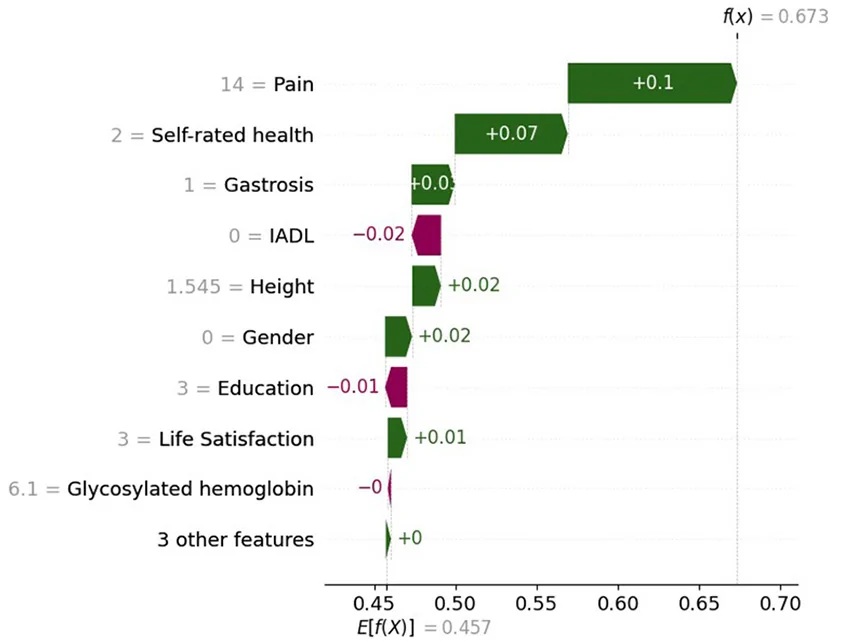
SHAP cascade plot of the depression risk prediction model for CKD patients.

To better interpret the model, we calculated the SHAP values for the model and created SHAP summary plots, SHAP dependence plots, and SHAP waterfall plots. Figure 5 demonstrates the contribution distribution of each feature to the model's predictions. Factors such as pain and self-rated health have a significant influence on the model's predictive output, and higher values of these features tend to increase the model's prediction of depression. In the dependence plots presented in Figure 6, more detail the specific impact of individual features on the model's predictive outcomes are elucidated. The waterfall plot, which displays the specific impact of a sample's features on the model's prediction, randomly selects a positive sample. The results show that pain and self-rated health are the main factors driving the increase in the predicted probability of depression, while IADLs and education level have a certain negative impact on the prediction. This is illustrated in Figure 7. These visualizations facilitate an intuitive comprehension of the model's decision-making mechanisms, elucidating feature contributions at both individualized prediction and holistic feature importance levels.

### 3.5 Development of web application for the model

**Figure 8:**
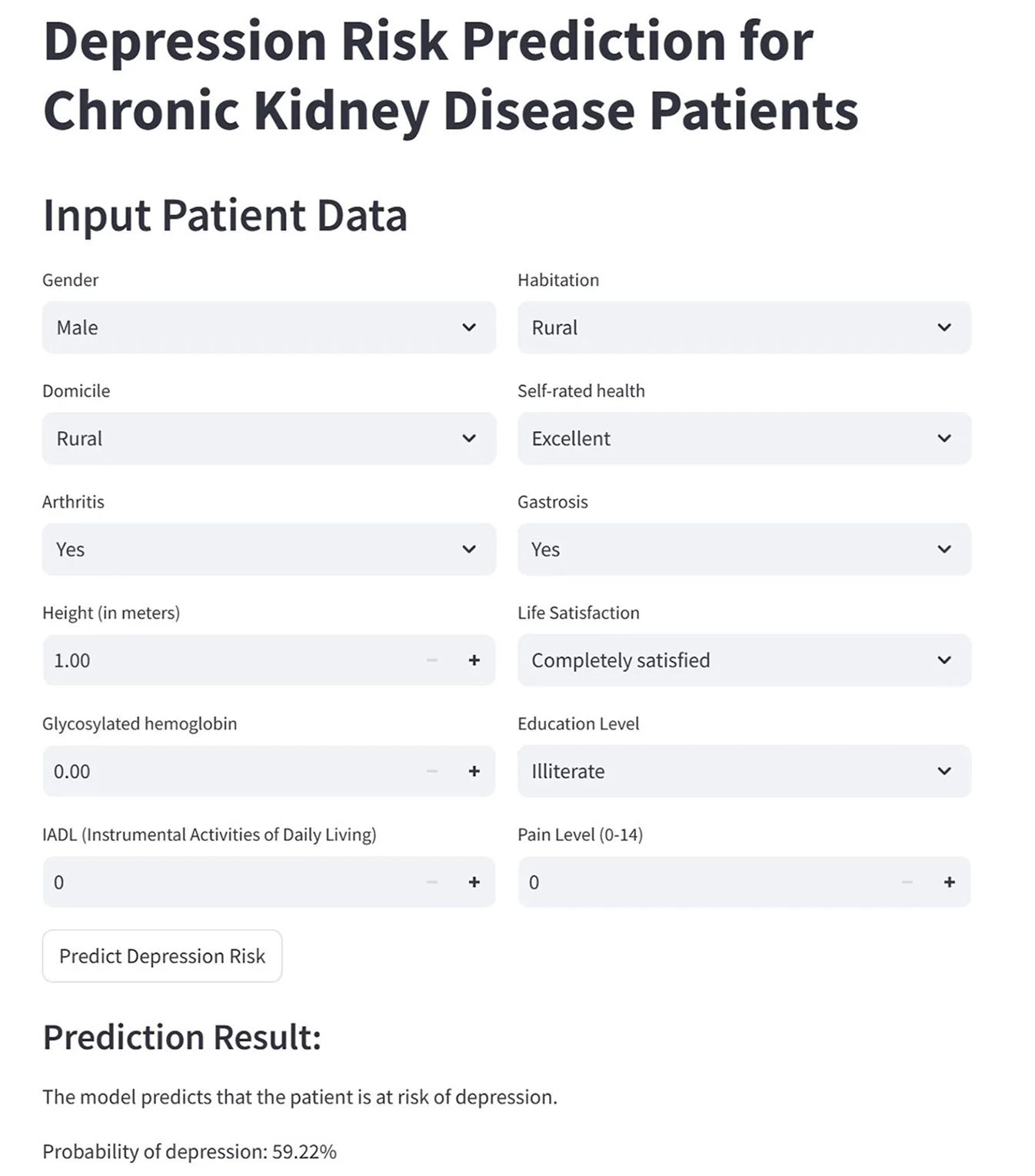
Web application interface of the depression risk prediction model for CKD patients.

To facilitate the application of the prediction model in clinical settings, we utilized Streamlit in Python to develop a corresponding web application. The web application developed in this study aims to provide clinicians with a rapid and convenient preliminary auxiliary screening tool for assessing depression risk, assisting in the identification of high-risk patients with chronic kidney disease. By inputting relevant features, users can obtain real-time prompts regarding their depression risk, thereby facilitating timely intervention or referral for further evaluation. It should be noted that this tool serves solely as an auxiliary screening instrument and does not possess diagnostic capabilities. Final diagnosis must rely on comprehensive clinical assessment and professional judgment. This is illustrated in Figure 8.

## 4 Discussion

This study indicates that the prevalence of depression among CKD patients is 44.4%, self-reported diagnosis may lead to under- or over-estimation of CKD prevalence, and future studies should adopt objective laboratory measurements to improve classification accuracy. Affected by the disease, the patient's physical function is weakened, which leads to the occurrence of negative psychological emotions such as depression (Zhang P. et al., 2025). A predictive model was established to estimate the risk of depression in CKD patients aged 45 and above in China. The LASSO method was employed for feature selection, and the random forest machine learning algorithm was utilized for the predictive task, with 12 key features ultimately developed and validated in the model. In the test model, the F1 score and AUC of the model were optimized, reaching an optimal state after 100 iterations. The SHAP analysis of the optimized predictive model identified influential features, such as pain, self-rated health, ADL, and education level. Additionally, the study demonstrated how each feature impacts the model's depression prediction. From a factor perspective, the selection of features or variables is crucial in developing a predictive model (Deo, 2024). This tool can quantify individual depression risk probability, assisting healthcare providers in rapidly identifying high-risk populations.

Notably, pain has been identified as a key predictor of depression in CKD patients. Those experiencing pain are more susceptible to developing depressive symptoms. Previous studies have consistently validated the association between pain and depression, with pain further established as a significant predictor of depressive symptoms (Hou, 2022). Research indicates that pain and depression may share analogous neurobiological mechanisms (Butska et al., 2025). Chronic pain can induce neurotransmitter imbalances in the brain—such as monoamine deficiency and dysregulation of the hypothalamic-pituitary-adrenal (HPA) axis—which are also implicated in depression. Clinical studies suggest that pain, as a stressor state, often triggers depression, with up to 85% of patients with pain experiencing severe depression (Aguera-Ortiz et al., 2011), and their coexistence can exacerbate the severity of both conditions (Sheng et al., 2017). Consequently, early diagnosis and management of pain through pharmacological and physical interventions are imperative. The provision of psychological counseling and emotional support is equally crucial to help patients cope with the mental distress associated with pain. In CKD patients, gastritis is associated with depression. The risk of depression in gastritis patients is influenced by the duration of the disease, abdominal pain, bloating, and Helicobacter pylori infection. Patients with a longer duration of gastritis are more prone to depression, possibly due to more frequent disease flare-ups and medical visits, leading to fears about disease progression and potential malignancy, increasing the risk of depression (Ma et al., 2023). Furthermore, the study revealed that CKD patients with lower levels of education are more susceptible to depression. Research has found that most patients with lower education levels have a vague understanding of the causes, progression, and prognosis of their disease, leading to excessive fear (Aydin et al., 2025). A negative disease thinking pattern can lead to serious biases when receiving health-related information, resulting in negative evaluations and cognition (Chen, 2015). Education level may affect CKD patients' disease perception, leading to cognitive biases and promoting the onset of depression. It is not only an independent risk factor for depression in CKD patients but also affects their quality of life (Ho et al., 2025). The results showed that gender is a predictor of depression. Women, as daughters, wives, and mothers in the family, often play more responsible and patient social roles and face work pressure. Due to CKD, they often cannot fulfill their previous family or social responsibilities, thus bearing more psychological burden (Zhang Y. et al., 2025). A study from Taiwan found that women undergoing hemodialysis are more susceptible to stress from physical symptoms and vascular issues (Yeh and Chou, 2007). Moreover, women are more likely to immerse themselves in negative emotions and develop depressive symptoms, while men prefer to actively process depressive emotions (Kim et al., 2017). Although the impact of the following factors on depression is less significant than the aforementioned predictors (Zhao et al., 2023), the study also found that household registration, place of residence, arthritis, glycated hemoglobin is also one of the factors affecting depression in CKD patients (Cheng et al., 2016). Notably, height likely serves as a proxy indicator of early-life nutritional status, socioeconomic background, or body physique, which are indirectly associated with mental health in later life. However, the underlying mechanisms likely involve multiple complex factors, including socioeconomic status and accessibility to medical resources, which require further research for validation.

To date, no predictive model specifically targeting depressive symptoms in patients with CKD has been reported. A notable innovation of this study lies in employing SHAP values to interpret the machine learning model. By calculating the SHAP values of various feature variables in the test dataset, we assessed their respective contributions to the predictive outcomes. The SHAP summary plot visually illustrates which features exert positive or negative effects on the predictions. Meanwhile, the feature importance plot reflects the average significance of each feature across the entire dataset. Furthermore, SHAP dependence plots reveal how different features influence the model's output at varying levels, thereby providing clearer insights into the specific impacts of factors such as gender, domicile, habitation, self-rated health, arthritis, gastric, height, life satisfaction, glycated hemoglobin levels, education level, IADL, and pain conditions on the prediction results. Through specific cases, the SHAP force plot demonstrates how different features interact collectively in individual risk predictions. Overall, this model enables quantitative evaluation of the influence of various factors on depression in CKD patients, thereby facilitating precise identification of individuals at potential risk and laying a solid foundation for developing targeted interventions and personalized prevention strategies. These efforts contribute to improving the quality of life for CKD patients and advancing the process of healthy aging. But the external validation or multi-center validation has not been conducted, thus caution is required when generalizing the findings to other populations. At present, the model is more suitable as an auxiliary screening tool for identifying high-risk individuals, rather than as a replacement for clinical diagnosis. Then, this study is a cross-sectional predictive classification model rather than a longitudinal prospective risk prediction model. Future prospective studies are needed to further validate its clinical utility. Moreover, in our subsequent prospective follow-up study, clinical implementation studies are needed to assess feasibility, usability, and impact on patient outcomes. We will further analyze the onset time of depression in CKD patients, explore the temporal correlation between CKD progression and depression onset, and verify the predictive value of the model for incident depression.

## 5 Conclusions

This study indicates that gender, household registration, place of residence, self-rated health, arthritis, stomach disease, height, life satisfaction, glycated hemoglobin, education level, IADL, and pain are risk factors for depression among patients with CKD. In summary, we have identified predictive variables highly associated with depression in middle-aged and elderly patients with CKD in China and constructed a predictive model based on these factors, the model is a cross-sectional auxiliary screening tool for identifying current depressive symptoms in CKD patients. Based on these risk factors, we can assess the probability of depression in CKD patients. Patients with CKD can intervene to reduce their risk of depression by addressing these risk factors. Clinical staff can use the predictive model to quickly identify CKD patients at higher risk of depression, facilitating early detection, early intervention, and early treatment of depression.

## Data Availability

The original contributions presented in the study are included in the article/supplementary material, further inquiries can be directed to the corresponding authors.
